# Occupational and environmental risk factors for chronic rhinosinusitis in China: a multicentre cross-sectional study

**DOI:** 10.1186/s12931-016-0366-z

**Published:** 2016-05-17

**Authors:** Wen-Xiang Gao, Chun-Quan Ou, Shu-Bin Fang, Yue-Qi Sun, Hua Zhang, Lei Cheng, Yan-Jun Wang, Dong-Dong Zhu, Wei Lv, Shi-Xi Liu, P. Z. Li, Geng Xu, Jianbo Shi, Qing-Ling Fu

**Affiliations:** Otorhinolaryngology Hospital, The First Affiliated Hospital, Sun Yat-sen University, 58 Zhongshan Road II, Guangzhou, Guangdong 510080 China; State Key Laboratory of Organ Failure Research, Department of Biostatistics, Guangdong Provincial Key Laboratory of Tropical Disease Research, School of Public Health, Southern Medical University, Guangzhou, Guangdong China; Department of Otorhinolaryngology, The First Affiliated Hospital, Xinjiang Medical University, Urumqi, Xinjiang China; Department of Otorhinolaryngology, The First Affiliated Hospital, Nanjing Medical University, Nanjing, Jiangsu China; Department of Otorhinolaryngology, Union Hospital, Tongji Medical College, Huazhong University of Science and Technology, Wuhan, Hubei China; Department of Otolaryngology Head and Neck, China-Japan Union Hospital, Jilin University, Changchun, Jilin China; Department of Otolaryngology, Peking Union Medical College Hospital, Beijing, China; Department of Otorhinolaryngology, West China Hospital of Medicine, Sichuan University, Chengdu, Sichuan China; Department of Otorhinolaryngology, Affiliated Huai’an First People’s Hospital, Nanjing Medical University, Huaian, Jiangsu China

**Keywords:** China, Chronic rhinosinusitis, Prevalence, Occupational and environmental risk factors

## Abstract

**Background:**

Chronic rhinosinusitis (CRS) is defined as a condition of inflammation in the paranasal sinus mucosa persisting for more than 12 weeks. We previously reported that the prevalence of CRS was about 8 % in China. Here, we aim to investigate the occupational and environmental risk factors associated with CRS.

**Methods:**

Data were collected from seven Chinese cities: Urumqi, Changchun, Beijing, Wuhan, Chengdu, Huaian and Guangzhou. CRS was diagnosed according to the European Position Paper on Rhinosinusitis and Nasal Polyps (EP^3^OS) document. Participants were asked to complete a standardized questionnaire, which was developed by the Global Allergy and Asthma European Network (GA^2^LEN) project and covered sociodemographic characteristics, CRS-related symptoms and occupational and environmental exposures. We evaluated the association between CRS and various occupational and environmental factors using odds ratios (ORs) and 95 % confidence intervals (95 % CIs).

**Results:**

The total study population consisted of 10,633 subjects, 850 (7.99 %) of whom were defined as having CRS according to the EP^3^OS criteria. We found that there were significant associations between occupational and environmental factors and CRS. Specifically, having a clearance-related job, occupational exposure to dust, occupational exposure to poisonous gas, a pet at home or carpet at home or at the workplace were risk factors for CRS. Additionally, the method used to keep warm in winter, the duration of time spent using air conditioning in summer and the frequency of exposure to mouldy or damp environments were significantly different in subjects with and without CRS.

**Conclusions:**

Our data showed that some occupational and environmental exposures are strongly associated with CRS, which aids in understanding the epidemiology of CRS.

## Background

Chronic rhinosinusitis (CRS), one of the most common chronic diseases, is defined as a condition of inflammation in the paranasal sinus mucosa persisting for more than 12 weeks. The prevalence of CRS has been reported to range from 6 to 27.1 % [1–5]. It is evident that CRS is associated with a substantially impaired quality of life [6], reduced workplace productivity and serious medical treatment costs [5, 7]. As the pathophysiology of this chronic condition has attracted high amounts of attention [8], an increasing number of studies [9, 10] have focused on potential risk factors associated with CRS. Two studies of the Korea National Health and Nutrition Examination Survey [11, 12] identified several risk factors for CRS, including influenza vaccination, septal deviation and allergic rhinitis. Moreover, there were significantly increased prevalence of chronic rhinosinusitis in plant and machinery operators and assemblers, craft and related trade workers and the unemployed. Trine Thilsing et al. [13] performed a cross-sectional survey of 3099 subjects in Denmark and found that occupational exposure to gases, fumes, dust or smoke was associated with a higher CRS prevalence. However, previous studies [1, 11, 12] have mostly focused on general risk factors, such as smoke exposure, or on general occupations or environments as opposed to specific exposures to occupational or environmental factors like air conditioning that may have important effects on disease risk.

We previously conducted a cross-sectional investigation in China and found that the prevalence of CRS was 8.0 % and varied with different sociodemographic subpopulations [2]. Notably, CRS was more prevalent in people with specific medical conditions, including allergic rhinitis, asthma, chronic obstructive pulmonary disease and gout. The present study further investigated the detailed occupational and environmental risk factors associated with CRS based on the dataset of this seven-city survey. The identification of risk factors is important for providing further guidance to prevent CRS.

## Methods

### Participants and sampling

A cross-sectional investigation was done in seven Chinese cities: Urumqi, Changchun, Beijing, Wuhan, Chengdu, Huaian and Guangzhou. These cities covered the North, Middle and South of China and have diverse climates and socioeconomics. The study protocol was approved by the Ethics Committee of The First Affiliated Hospital, Sun Yat-sen University (the principle centre), China. The interviewers explained the purpose of the investigation and the procedures and acquired the informed consent of all subjects involved in the study before conducting the interviews.

We used a stratified four-stage random sampling method to select the participants. For each city (the strata), based on the list of administrative districts, streets and communities, the coordinator of this study randomly sampled two districts and subsequently used simple random sampling to select two streets within each selected district and two communities from each selected street. Fifty-six communities (eight communities in each city) were selected. In the final stage, the investigators in each city performed cluster sampling. Approximately 65 households from each community were randomly sampled according to the door numbers provided by the local communities. The target subjects were all Chinese residents of chosen households who had lived in the local region for at least one year at the time of the survey.

### Instruments and data collection

The participants were interviewed face-to-face in their houses and were asked to complete a standardized questionnaire. The questionnaire was modified by the Global Allergy and Asthma European Network (GA^2^LEN) project and covered sociodemographic characteristics, CRS-related symptoms and occupational and environmental exposures. Participants were asked whether they engaged in a clearance-related or health care-related job and about their exposure level to dust, poisonous gas, carpet or mouldy or damp environments. Questions regarding participants’ methods of cooking and keeping warm in winter and the frequency of air conditioner use were also included in this questionnaire and considered to be important components of environmental exposures. According to the European Position Paper on Rhinosinusitis and Nasal Polyps (EP^3^OS) document [14], the definition of CRS is based on the presence of two or more of the following symptoms and at least one of the first two symptoms being present for more than 12 weeks in the last year: nasal obstruction/blockage/congestion, nasal discharge (anterior/posterior/nasal drip or purulent throat mucus), facial (forehead/nasal/eye) pain/pressure or a reduction in or loss of sense of smell. In principle, the questionnaire was self-administered, but ghostwriting by the parents was allowed for children, and assistance from trained interviewers was provided for illiterate participants and for clarification of the questions.

### Data analysis

All data analyses were performed using IBM SPSS 20.0. Characteristics of the CRS group and non-CRS group were compared using a χ2 test or Mann–Whitney test depending on the nature of the factor under consideration. A difference was regarded as statistically significant if the two-tailed P value was less than 0.05. We evaluated the association between CRS and various occupational and environmental factors using odds ratios (ORs) and the corresponding 95 % confidence intervals (95 % CIs). In addition, we performed multivariate logistic regression analyses and presented the adjusted ORs after controlling for sociodemographic characteristics and smoking.

## Results

### Comparisons of sociodemographic characteristics between the two groups

The total study population consisted of 10,633 subjects, 851 (7.99 %) of whom were defined as having CRS according to the EP^3^OS criteria, and the response rate of the total study population was 87 % [2]. We previously reported [2] that the overall prevalence of CRS was 8.00 % in the general population, though some geographic variations were observed. This study further analysed the sociodemographic characteristics of the subjects with and without CRS, as shown in Table 1. Our data showed that gender was a significant factor affecting the prevalence of CRS (*P* = 0.005). Moreover, ethnicity (Han or minority), marital status (married, divorced, widowed, unmarried) and educational attainment were also significantly different between non-CRS and CRS subjects, while age and household monthly income per person were not.

**Table 1:** Comparisons of sociodemographic characteristics between the two groups

Factors	Groups	Non-CRS (%)	CRS (%)	*P*-value
Gender	Male	4685(47.9)	450(52.9)	0.005
	Female	5098(52.1)	400(47.1)	
Age group	0 ~ 14 years	603(6.2)	41(4.8)	0.484
	15 ~ 34 years	2856(29.2)	280(33.0)	
	35 ~ 59 years	4466(45.7)	368(43.3)	
	> = 60 years	1845(18.9)	160(18.8)	
Ethnicity	Han	9277(95.1)	788(92.7)	0.002
	Minority	475(4.9)	62(7.3)	
Marital status	Married	7115(73.0)	575(67.8)	0.001
	Divorced	126(1.3)	28(3.3)	
	Widowed	393(4.0)	38(4.5)	
	Unmarried	2115(21.7)	207(24.4)	
Educational attainment	Illiterate or primary	1492(15.3)	112(13.2)	0.010
	Secondary school	2038(20.9)	148(17.4)	
	High school	3100(31.7)	294(34.6)	
	College	2938(30.1)	280(33.0)	
	Masters or above	205(2.1)	15(1.8)	
Household monthly income per person	<RMB $1000	1143(11.7)	98(11.6)	0.281
	RMB $1001–3000	6010(61.5)	540(63.9)	
	>RMB $3000	2612(26.7)	207(24.5)

**Table 2:** Univariate analysis of the association between occupational and environmental factors and CRS

	Non-CRS n(%)	CRS n(%)	OR(95 % CI)	*P*-value
Clearance-related job	239(2.5)	32(3.9)	1.56(1.07,2.27)	0.020
Health care-related job	489(5.2)	47(5.7)	1.11(0.82,1.51)	0.509
Occupational exposure to dust	542(5.6)	101(12.0)	2.32(1.85,2.91)	0.001
Occupational exposure to poisonous gas	255(2.6)	58(6.9)	2.75(2.05,3.69)	0.001
Any pet at home	1121(13.4)	154(19.8)	1.60(1.32,1.92)	0.001
Any large piece of carpet at home	540(6.5)	92(11.9)	1.95(1.54,2.46)	0.001
Any carpet at workplace	89(1.1)	51(6.6)	6.55(4.60,9.32)	0.001
Method used to cook at home				
Electric or centralized heating	9352(96.1)	814(96.3)	1	0.559
Coal	149(1.5)	15(1.8)	1.16(0.68,1.97)	
Firewood	234(2.4)	16(1.9)	0.79(0.47,1.31)	
Method of keeping warm in winter				
Electric or centralized heating	6380(96.4)	521(94.2)	1	0.003
Coal	137(2.1)	1313(2.4)	1.16(0.65,2.07)	
Firewood/charcoal	100(1.5)	19(3.4)	2.33(1.41,3.83)	
Use of air conditioning in summer				
< 3 days/week	5154(60.7)	439(56.6)	1	0.016*
3–6 days/week	2137(25.2)	208(26.8)	1.14(0.96,1.36)	
Everyday	1200(14.1)	129(16.6)	1.26(1.03,1.55)	
Exposure to mouldy or damp environments				
Never	7111(85.2)	593(76.5)	1	0.001*
Occasionally	1021(12.2)	137(17.7)	1.61(1.32,1.96)	
Frequently or everyday	219(2.7)	45(5.8)	2.46(1.77,3.43)	

*Score test for trend of odds

**Table 3:** Multivariate analysis of the association between occupational and environmental factors and CRS

	Adjusted OR (95 % CI)^a^	
	Full model	Stepwise model
Clearance-related job	1.93(1.11,3.38)	1.90(1.09,3.29)
Health care-related job	0.60(0.38,0.96)	-
Occupational exposure to dust	2.22(1.58,3.12)	2.21(1.58,3.10)
Occupational exposure to poisonous gas	1.82(1.15,2.88)	1.77(1.12,2.80)
Any pet at home	1.17(0.89,1.52)	-
Any carpet at home	1.50(1.05,2.13)	1.71(1.25,2.33)
Any carpet at workplace	3.70(2.26,6.05)	3.76(2.32,6.09)
Method of cooking at home (reference group: electric or gas)		
Coal	0.84(0.41,1.72)	-
Firewood	0.79(0.37,1.71)	-
Method of keeping warm in winter (reference group: electric or centralized heating)		
Coal	1.12(0.59,2.14)	1.06(0.58,1.94)
Firewood/charcoal	2.19(1.21,3.95)	2.04(1.18,3.52)
Frequency of using air conditioning in summer (reference group: <3 days/week)		
3–6 days/week	1.16(0.92,1.46)	1.16(0.92,1.45)
Everyday	1.38(1.02,1.87)	1.38(1.02,1.87)
Exposure to mouldy or damp environments (reference group: never)		
Occasionally	1.50(1.13,2.01)	1.49(1.12,1.98)
Frequently or everyday	2.41(1.50,3.85)	2.37(1.48,3.79)

^a^Data were odds ratios (ORs) adjusted for sociodemographic variables (age, gender, marital status, education and household income per person), smoking and other factors in this table

### Univariate analysis of the association between occupational and environmental factors and CRS

The univariate analyses utilized to examine the effects of some detailed risk factors on the prevalence of CRS revealed some risk factors which possibly affect CRS prevalence (Table 2). We found that having a clearance-related job (OR, 1.56; 95 % CI: 1.07–2.27), occupational exposure to dust (OR, 2.32; 95 % CI: 1.85–2.91), occupational exposure to poisonous gas (OR, 2.75; 95 % CI: 2.05–3.69), a pet at home (OR, 1.60; 95 % CI: 1.32–1.92), carpet at home (OR, 1.95; 95 % CI: 1.54–2.46) or at the workplace (OR, 6.55; 95 % CI: 4.60–9.32), and the method used to cook at home (electric or centralized heating, coal, firewood/charcoal) were significant risk factors for CRS (Table 2). Moreover, the method used to keep warm in winter, the duration of time spent using air conditioning in summer and the frequencies of exposure to mouldy or damp environments were significantly different in subjects with and without CRS. Furthermore, a trend test revealed a significant dose–response relationship with the risk of CRS increasing with the duration of time spent using air conditioning in summer and with the frequencies of exposure to mouldy or damp environments (*P* = 0.016, 0.001, respectively). However, there was no significant difference in the proportion of health care-related jobs or the method used to cook at home in subjects with and without CRS (*P* = 0.509, 0.559, respectively).

### Multivariate analysis of the association between occupational and environmental factors and CRS

After adjusting for smoking and all the sociodemographic factors shown in Table 1 (age, gender, marital status, education and household income per person), there were still some significant environmental and occupational factors associated with CRS, including having a clearance-related job (adjusted OR, 1.90; 95 % CI: 1.09–3.29), occupational exposure to dust (adjusted OR, 2.21; 95 % CI: 1.58–3.01) or poisonous gas (adjusted OR, 1.77; 95 % CI: 1.12–2.80) and having carpet at home (adjusted OR, 1.71; 95 % CI: 1.25–2.33) or at the workplace (adjusted OR, 3.76; 95 % CI: 2.23–6.09) (Table 3). Moreover, using firewood/charcoal to keep warm in winter, using air conditioning daily in summer and being exposed to mouldy or damp environments were significantly associated with an increased likelihood of CRS (Table 3). Interestingly, having a pet at home was not significantly associated with CRS after adjusting for other factors in the multiple logistic regression analysis (Table 3).

## Discussion

We found a systematic review about occupational and environmental risk factors for chronic rhinosinusitis [15] in PubMed that was published in May 2015. In this review, 41 studies met their inclusion criteria. These studies were published between 1964 and 2012 and were performed in 14 different countries. Thirty-seven of the studies evaluated only occupational risk factors, one investigated only environmental risk factors, and three included both types of exposures. Of the 41 papers included, 11 met the probable CRS criteria, eight met the possible criteria, and 22 met the least likely CRS criteria. We further used the key words “occupational and environmental risk factors, chronic rhinosinusitis; Date 2012 to present” to search articles in PubMed and found only one study meeting the criteria: “Chronic rhinosinusitis and occupational risk factors among 20- to 75-year-old Danes-A GA^2^LEN-based study” [13], which was conducted by Trine Thilsing et al. in Denmark. This study was performed in the summer of 2008, meaning that the definition of CRS it used was based on the EP^3^OS 2007 criteria [4]. Therefore, it may be that since the EP^3^OS 2012 CRS definition [14], a new and more accurate diagnosis definition for CRS, was published, there have been no prevalence studies regarding the occupational and environmental risk factors for CRS except for the current study.

The EP^3^OS 2012 [14] indication proposed clear guidelines for defining rhinosinusitis that can be applied well to epidemiological and clinical research. The questions used in the GA^2^LEN questionnaire for CRS were based on the EP^3^OS 2012 definition for rhinosinusitis. The questionnaire used in our study was further modified from the GA^2^LEN questionnaire by adding questions regarding some risk factors for CRS.

Previous studies found that smoking and allergies are potent risk factors for CRS. Chen et al. [16] found a significant association between current smoking and rhinosinusitis in women but not men. Lieu and Feinstein [17] revealed a 20 % increased risk of rhinosinusitis in current smokers. Lotvall J, et al. [18] reported that multi-symptom asthma was closely related to symptoms of nasal allergy as well as to CRS. In our cross-sectional investigation, we reported [2] that having asthma or a nasal allergy significantly increased the risk of CRS, which was consistent with some studies discovering positive associations between sinus disease or CRS and asthma [19–23]. Beside the living habits and related diseases, we found that some occupational and environmental exposures were the risk factors for CRS.

Occupational factors are very complicated and have the potential to affect the prevalence of CRS. Koh and his group used data from the 1998, 2001 and 2005 Korea National Health and Nutrition Examination Surveys (KNHANES) to compare the CRS prevalence across the major groups of the standard occupational classification with clerical workers as the reference group [12]. They found that the CRS prevalence was significantly increased among plant or machine operators and assemblers, those with elementary occupations, craft and related trades workers and the unemployed. Occupational exposures to dust, fumes and gases were found to increase the prevalence of rhinosinusitis by comparing the prevalence of rhinosinusitis in some “high exposure” occupations such as spice factory workers, paper-recycling workers, cement factory workers and wool textile workers to that among “low exposure” occupations such as fruit-bottling workers, packers in the food industry, health examiners and delivery workers [24–27]. Furthermore, Hox et al. [28] and Fokkens WJ, Lund VJ [14], Mullol J, et al. [29] found that occupational risk factors may be related to difficult-to-treat CRS cases. This result may be consistent with our finding that occupational exposures to dust, poisonous gas or mouldy or damp environments were risk factors for CRS and may even lead to aggravate. However, we did not find that the effects statistically increased with the exposure level to dust or poisonous gas, probably because of a small sample size of subgroups for different exposure levels in the general population. Further data of the dose and duration of the exposure collected from specific occupational workers should be done to identify the potential dose–response relationship. In our study, we found that having a clearance-related job was a risk factor for CRS in both univariate and multivariate analyses. Conversely, having a health care-related job did not correlate with CRS incidence. Occupational exposures to dust and poisonous gases were high risk factors for CRS, because both the unadjusted and adjusted ORs for these factors were higher than 2. These results indicate that a clean occupational environment with less required physical activity can reduce the risk of CRS.

Few studies about the effect of environment exposures to CRS were reported. A recent system review [15] on the occupational and environmental risk factors for chronic rhinosinusitis showed there were only 4 studies about the effect of environmental exposures to CRS and none of them were held in China. Previous studies mostly focused on smoking [16, 17, 20] and poor air quality [30]. We also reported the effects of smoking on the prevalence of CRS [2]. Here we focused on relationship between the living environmental exposure and the prevalence of CRS. We found that animal fur may be important trigger of CRS. The univariate analysis of the data indicated that people who have a pet at home or carpet at home or in the workplace were more likely to suffer from CRS than individuals without these factors. After adjusting for sociodemographic variables and smoking, the OR values for these factors were still higher than 1. Having carpets has also been found to be a risk factor of allergic rhinitis in many studies. Furthermore, the duration of time spent using air conditioning in summer and frequencies of exposures to mouldy or damp environments were found to have a dose-dependent relationship with the prevalence of CRS. Interestingly, we found that using firewood or charcoal to keep warm in winter(OR = 2.33, 95 % CI 1.41–3.83) may be a risk factor to CRS but using firewood to cook at home(OR = 0.79, 95 % CI 0.47–1.31) may not. This may because wood is incompletely combusted and easily produces carbon monoxide if it issued for heating. However, the wood is completed combusted if it is used for cook. Bhattacharyya N [31] reported that CO was a risk factors to CRS. This is a new risk factor for CRS. A significant correlation between CRS and a dusty or dirty environment was found. Our findings suggest that cleaner occupational and living environments may help to reduce the prevalence of CRS. Cleaning carpets and using air conditioners less frequently may be useful to prevent the development of CRS. Wearing masks in an environment containing dust or poisonous gas may also help to reduce the risk of CRS. Our study provided some guidance as to how to provide healthier work or living environments, and we believe that this guidance will further increase quality of life and decrease the economic burden of CRS.

There are some limitations associated with this study. First of all, because objective nasal examinations could not be taken in a large household survey, all outcome and exposure measures were self-reported. There may be recall bias and misclassification, probably leading to underestimate the effects on CRS in our study. We used a strict epidemiological diagnosis criteria and the questionnaire was modified according to the EP^3^OS 2012 to reduce the potential bias. Secondly, we only investigated the relationship between CRS and clearance-related jobs and health-care related jobs, while other jobs such as administrator, technician or worker were not included in our study. At last, further cohort studies or experimental studies may help to understand the biological mechanism underlying the associated factors.

## Conclusion

Our study identified occupational and environmental exposures with positive correlations to the development of CRS, which might facilitate a better understanding of the epidemiology of CRS and provide important information for CRS prevention.

## References

[1] Hastan D, Fokkens WJ, Bachert C, Newson RB, Bislimovska J, Bockelbrink A, Bousquet PJ, Brozek G, Bruno A, Dahlen SE (2011). Chronic rhinosinusitis in Europe--an underestimated disease. A GA(2)LEN study. Allergy.

[2] Shi JB, Fu QL, Zhang H, Cheng L, Wang YJ, Zhu DD, Lv W, Liu SX, Li PZ, Ou CQ, Xu G: Epidemiology of chronic rhinosinusitis: results from a cross-sectional survey in seven Chinese cities. Allergy. 2015;70:533-539. 10.1111/all.12577 PMC4409092 25631304

[3] Pilan RR, Pinna FR, Bezerra TF, Mori RL, Padua FG, Bento RF, Perez-Novo C, Bachert C, Voegels RL (2012). Prevalence of chronic rhinosinusitis in Sao Paulo. Rhinology.

[4] Fokkens W, Lund V, Mullol J, European Position Paper on R, Nasal Polyps g: European position paper on rhinosinusitis and nasal polyps 2007. Rhinol Suppl 2007:1-136. 17844873

[5] Hamilos DL. Chronic rhinosinusitis: epidemiology and medical management. J Allergy Clin Immunol. 2011;128:693-707; quiz 708-699. 10.1016/j.jaci.2011.08.004 21890184

[6] Fu QL, Ma JX, Ou CQ, Guo C, Shen SQ, Xu G, Shi J. Influence of self-reported chronic rhinosinusitis on health-related quality of life: a population-based survey. PLoS One. 2015;10:e0126881. 10.1371/journal.pone.0126881 PMC4433264 25978550

[7] Halawi AM, Smith SS, Chandra RK. Chronic rhinosinusitis: epidemiology and cost. Allergy Asthma Proc. 2013;34:328-34. 10.2500/aap.2013.34.3675 23883597

[8] Van Crombruggen K, Zhang N, Gevaert P, Tomassen P, Bachert C. Pathogenesis of chronic rhinosinusitis: inflammation. J Allergy Clin Immunol. 2011;128:728-32. 10.1016/j.jaci.2011.07.049 21868076

[9] Smith DF, Ishman SL, Tunkel DE, Boss EF. Chronic rhinosinusitis in children: race and socioeconomic status. Otolaryngol Head Neck Surg. 2013;149:639-44. 10.1177/0194599813498206 23884283

[10] Soler ZM, Mace JC, Litvack JR, Smith TL. Chronic rhinosinusitis, race, and ethnicity. Am J Rhinol Allergy. 2012;26:110-16. 10.2500/ajra.2012.26.3741 PMC3345896 22487286

[11] Kim YS, Kim NH, Seong SY, Kim KR, Lee GB, Kim KS. Prevalence and risk factors of chronic rhinosinusitis in Korea. Am J Rhinol Allergy. 2011;25:117-121. 10.2500/ajra.2011.25.3630 21679523

[12] Koh DH, Kim HR, Han SS. The relationship between chronic rhinosinusitis and occupation: the 1998, 2001, and 2005 Korea National health and nutrition examination survey (KNHANES). Am J Ind Med. 2009;52:179-84. 10.1002/ajim.20665 19051236

[13] Thilsing T, Rasmussen J, Lange B, Kjeldsen AD, Al-Kalemji A, Baelum J. Chronic rhinosinusitis and occupational risk factors among 20- to 75-year-old Danes-A GA(2) LEN-based study. Am J Ind Med. 2012;55:1037-1043. 10.1002/ajim.22074 22648974

[14] Fokkens WJ, Lund VJ, Mullol J, Bachert C, Alobid I, Baroody F, Cohen N, Cervin A, Douglas R, Gevaert P, et al. European Position Paper on Rhinosinusitis and Nasal Polyps 2012. Rhinol Suppl 2012:3 p preceding table of contents, 1-298. 22764607

[15] Sundaresan AS, Hirsch AG, Storm M, Tan BK, Kennedy TL, Greene JS, Kern RC, Schwartz BS. Occupational and environmental risk factors for chronic rhinosinusitis: a systematic review. Int Forum Allergy Rhinol. 2015;5:996-1003. 10.1002/alr.21573 PMC4681694 26077513

[16] Chen Y, Dales R, Lin M: The epidemiology of chronic rhinosinusitis in Canadians. Laryngoscope. 2003;113:1199-205. 10.1097/00005537-200307000-00016 12838019

[17] Lieu JE, Feinstein AR. Confirmations and surprises in the association of tobacco use with sinusitis. Arch Otolaryngol Head Neck Surg. 2000;126:940-46. 10.1001/archotol.126.8.940 10922224

[18] Lotvall J, Ekerljung L, Lundback B (2010). Multi-symptom asthma is closely related to nasal blockage, rhinorrhea and symptoms of chronic rhinosinusitis-evidence from the West Sweden Asthma Study. Respir Res.

[19] Dunlop G, Scadding GK, Lund VJ (1999). The effect of endoscopic sinus surgery on asthma: Management of patients with chronicrhinosinusitis, nasal polyposis, and asthma. Am J Rhinol.

[20] Ikeda K, Tanno N, Tamura G, Suzuki H, Oshima T, Shimomura A, Nakabayashi S, Takasaka T. Endoscopic sinus surgery improves pulmonary function in patients with asthma associated with chronic sinusitis. Ann Otol Rhinol Laryngol. 1999;108:355-59. 10.1177/000348949910800407 10214782

[21] Leynaert B, Neukirch F, Demoly P, Bousquet J. Epidemiologic evidence for asthma and rhinitis comorbidity. J Allergy Clin Immunol. 2000;106:S201-205. 10.1067/mai.2000.110151 11080732

[22] ten Brinke A, Grootendorst DC, Schmidt JT, De Bruine FT, van Buchem MA, Sterk PJ, Rabe KF, Bel EH. Chronic sinusitis in severe asthma is related to sputum eosinophilia. J Allergy Clin Immunol. 2002;109:621-26. 10.1067/mai.2002.122458 11941310

[23] Bresciani M, Paradis L, Des Roches A, Vernhet H, Vachier I, Godard P, Bousquet J, Chanez P. Rhinosinusitis in severe asthma. J Allergy Clin Immunol. 2001;107:73-80. 10.1067/mai.2001.111593 11149994

[24] Zuskin E, Mustajbegovic J, Schachter EN, Kanceljak B, Kern J, Macan J, Ebling Z. Respiratory function and immunological status in paper-recycling workers. J Occup Environ Med. 1998;40:986-93. 10.1097/00043764-199811000-00009 9830606

[25] Zuskin E, Skuric Z, Kanceljak B, Pokrajac D, Schachter EN, Witek TJ. Respiratory findings in spice factory workers. Arch Environ Health. 1988;43:335-39. 10.1080/00039896.1988.9934944 3178290

[26] Al-Neaimi YI, Gomes J, Lloyd OL. Respiratory illnesses and ventilatory function among workers at a cement factory in a rapidly developing country. Occup Med (Lond). 2001;51:367-73. 10.1093/occmed/51.6.367 11584114

[27] Zuskin E, Mustajbegovic J, Schachter EN, Kanceljak B, Godnic-Cvar J, Sitar-Srebocan V. Respiratory symptoms and lung function in wool textile workers. Am J Ind Med. 1995;27:845-57. 10.1002/ajim.4700270608 7645578

[28] Hox V, Delrue S, Scheers H, Adams E, Keirsbilck S, Jorissen M, Hoet PH, Vanoirbeek JA, Nemery B, Hellings PW. Negative impact of occupational exposure on surgical outcome in patients with rhinosinusitis. Allergy. 2012;67:560-65. 10.1111/j.1398-9995.2011.02779.x 22229752

[29] Batra PS, Tong L, Citardi MJ. Analysis of comorbidities and objective parameters in refractory chronic rhinosinusitis. Laryngoscope. 2013;123 Suppl 7:S1-11. 10.1002/lary.24418 24122826

[30] Min YG, Jung HW, Kim HS, Park SK, Yoo KY. Prevalence and risk factors of chronic sinusitis in Korea: results of a nationwide survey. Eur Arch Otorhinolaryngol. 1996;253:435-39. 10.1007/BF00168498 8891490

[31] Bhattacharyya N. Air quality influences the prevalence of hay fever and sinusitis. Laryngoscope. 2009;119:429-33. 10.1002/lary.20097 19160400

